# Body satisfaction and body weight in under- and healthy-weight adolescents: mediating effects of restrictive dieting, healthy and unhealthy food intake

**DOI:** 10.1007/s40519-018-0496-z

**Published:** 2018-03-08

**Authors:** Karolina Zarychta, Carina K. Y. Chan, Magdalena Kruk, Aleksandra Luszczynska

**Affiliations:** 1 Wroclaw Faculty of Psychology, SWPS University of Social Sciences and Humanities, 30b Ostrowskiego Street, 53-238 Wroclaw, Poland; 2 School of Psychology, Australian Catholic University, Banyo, QLD 4014 Australia; 3 University of Colorado at Colorado Springs, 1420 Austin Bluffs Pkwy, Colorado Springs, CO 80933-7105 USA

**Keywords:** Body satisfaction, Body weight, Healthy eating, Unhealthy eating, Restrictive dieting, Adolescence

## Abstract

**Purpose:**

Theoretical models, such as the transdiagnostic model of eating disorders highlight the role of cognitive factors (e.g., the way people perceive their bodies) and their associations with maladaptive weight management behaviors resulting in underweight. This paper aims at testing the indirect association of adolescent’s body satisfaction and body mass index (BMI) through restrictive dieting, healthy eating or unhealthy eating as well as moderating role of adolescent’s weight status.

**Methods:**

The study was conducted in 16 public middle and high schools in Central and Eastern Poland. A sample of 1042 under- and healthy-weight white adolescents aged 13–20 (BMI: 12.63–24.89) completed two self-reported questionnaires (fruit, vegetable, and energy-dense food intake) with a 11-month interval. Weight and height were measured objectively. Multiple mediation analysis and moderated multiple mediation analysis were conducted to test the study hypotheses.

**Results:**

Adolescents less satisfied with their bodies were more likely to diet restrictively and at the same time ate more unhealthy energy-dense food rather than healthy food, which in turn predicted lower BMI. No moderating effects of weight status were found.

**Conclusions:**

Low body satisfaction is a risk for restrictive diet and unhealthy food intake. Prevention programs may target under- and healthy-weight adolescents who are highly dissatisfied with their bodies, have a high intake of energy-dense food and apply a restrictive diet at the same time.

**Level of evidence:**

Level III: longitudinal cohort study.

## Introduction

Healthy body weight reduces the risk of heart disease, diabetes, high blood pressure, osteoarthritis and other related health problems [1]. On the other hand, underweight (as well as overweight and obesity) might affect adolescents’ physical and mental health [2], and lead to eating disorders (EDs) (as shown in Zarychta, Mullan, Kruk and Luszczynska [3]) or the development of EDs symptoms that do not meet the diagnostic criteria [4, 5]. Therefore, the identification of modifiable risk factors for underweight and factors promoting healthy body weight is of key importance in the prevention of their consequences in the future.

Theoretical models (e.g., transdiagnostic model of EDs) [6] highlight the role of cognitive factors in explaining eating behaviors and body weight. They focus primarily on the way people perceive their bodies, the contents of their thoughts, and perceptions of body weight and shape. The transdiagnostic model of EDs emphasizes that people let their outer appearance affect their self-evaluations. This leads to an excessive concentration on body weight and shape, and to maladaptive weight management behaviors (i.e., restrictive dieting, compulsive overeating, extremely healthy eating) resulting in underweight.

Body weight perception is one of the key cognitive factors emphasized as a significant determinants of both nutrition behaviors (e.g., Chen et al. [7]) and body mass index (BMI) [8]. Thus, it is important to explore the influence of psychological variables on diet, as they may help promote the uptake and maintenance of healthy behaviors in this population [9, 10]. Body weight-related perceptions (e.g., body satisfaction) are most often tested in over- and underweight groups. However, they are important for adolescents in general, including those with normal body weight, as unfavorable shifts in body weight perceptions may contribute to a development of unfavorable physical and mental health outcomes [11]. The present study fills that gap by investigating body satisfaction in normal weight adolescents with reference to underweight adolescents.

It has been well documented in previous studies that body satisfaction (defined as evaluation of one’s body) and its lower level (which represents body dissatisfaction) [12] is one of the main variables associated with not only EDs [13], but also with body weight status and body mass index [11]. Lower body satisfaction (conceptualized as the equivalent of higher body dissatisfaction in this study) is also linked to other health-related determinants and outcomes including lower self-esteem [14], anxiety and depression [15] or to subclinical eating pathologies [16] and various weight control behaviors [17]. On the other hand, higher body satisfaction is associated with individuals being less likely to diet restrictively or use other weight control behaviors, and with higher frequency of physical activity [18].

Among adolescents with normal weight or underweight, lower body satisfaction leads to lower engagement in healthy eating or excessive physical activity, which in turn leads to weight loss resulting in becoming underweight or underweight maintenance [19]. Thus, research is needed to explore whether body satisfaction predicts healthy or unhealthy eating behaviors, which in turn predicts unfavorable changes in BMI (i.e., body mass reduction) in the group of under- and healthy-weight adolescents. The present study will shed some light on this issue.

Physical appearance (including e.g., weight, height and body fat percentage) significantly changes in adolescence and it becomes especially big concern for teenage girls [20]. On the other hand, lower body satisfaction related to drive for muscularity can also be found in teenage boys [21]. In particular, both females and males want to have a lean and low in body fat physiques, but males additionally desire muscular bodies (e.g., Field et al. [22]). Thus, lower body satisfaction is a growing challenge in adolescences of both genders.

Results of several cross-sectional [13, 23] and longitudinal studies [24, 25] confirmed associations of lower body satisfaction with eating pathologies such as restrictive dieting, which may lead to underweight and/or EDs. On the other hand, very few of these studies clarified the possible mechanism through which body satisfaction may explain body weight. One probable pathway is that if the level of body satisfaction constitutes a risk factor for low levels of healthy eating behaviors which in turn predicts lower healthy body weight. Moreover, most studies focused on restrictive or binge eating but whether the food intake was healthy or not (accounting for high intake of fruit and vegetable, and reducing energy-dense foods or high intake of energy-dense foods) was overlooked. The present study investigated both heathy eating and eating pathologies indicators, such as restrictive dieting or eating energy-dense foods (unhealthy eating).

The majority of previous research referred to general population of adolescents [26] or focused on overweight and obese adolescents [27, 28] suggesting that promoting body satisfaction might be most beneficial for weight loss or maintaining healthy body weight. However, it can be assumed that an investigation of the associations among body satisfaction, healthy and unhealthy eating, and BMI should account also for under- and healthy-weight adolescents.

Furthermore, to fully understand temporal effects among body satisfaction, healthy eating or unhealthy dieting and body mass index (BMI), self-reported variables should be measured at separate time points [29]. Unfortunately, most of research so far has mostly included one measurement point [30–32]. The hypotheses in the present study will be tested using two measurement points to establish temporal precedence.

The aim of this prospective study was to investigate the associations between body satisfaction, healthy and unhealthy eating, restrictive dieting, and adolescents’ BMIs in a non-clinical sample of adolescents with underweight or healthy-weight. We hypothesized that the long-term indirect effects of body satisfaction on adolescent’s BMI will go rather through restrictive dieting than through either healthy or unhealthy eating. In particular, we hypothesized that:

- the indirect effect of body satisfaction (Time 1; T1) on BMI (T2) would be mediated by healthy eating (Time 2; T2); (Hypothesis 1; H1);
- the indirect effect of body satisfaction (T1) on BMI (T2) would be mediated by unhealthy eating (T2) (H2);
- the indirect effect of body satisfaction (T1) on BMI (T2) would be mediated by restrictive dieting (T2) (H3);
- body satisfaction (T1) associations with healthy eating (T2), unhealthy eating (T2) and restrictive dieting (T2) would be moderated by body weight status (1—underweight, 2—normal weight) (H4).

As previous research established that sex [33, 34], age [35] and BMI [36] may be relevant determinants of the cognitive and behavioral ED risk factors, all three hypotheses were tested accounting for the effects of sex, age and BMI (T1) on the dependent variable, that is BMI at T2.

## Method

### Participants

At Time 1 (T1; baseline), 1297 adolescents (58.2% girls) aged 13–20 years (M 16.54, SD 0.90) with BMIs ranging from 12.63 to 41.21 (M 22.30, SD 3.77) participated in the study, of whom 33 (2.6%) adolescents were underweight, 888 (69.1%) had normal body weight, 257 (19.8%) were overweight, and 107 (8.2%) obese (calculated according to WHO growth reference for children and adolescents; de Onis et al. [37]). At Time 2 (T2; 11 months later), a total of 911 (52.3% girls) adolescents aged 14–20 years (M 17.19, SD 0.93) with BMIs ranging from 14.24 to 39.66 (M 22.13, SD 3.97) participated in the study. At T2, 33 (3.6%) participants were underweight, 681 (74.8%) had normal body weight, 145 (15.9%) were overweight, 52 (5.7%) or obese. Overweight (*n* = 257) and obesity (*n* = 107) were identified as an exclusion criterion in the study, since the mechanisms related to their higher weight might be different than in the groups with underweight or normal weight. All participants were white. The majority (64%) lived in urban areas, with 36% living in rural areas.

The total attrition rate was 29.8%. Missing data from those who dropped out at T2 were imputed. Therefore, data collected from *N* = 1042 adolescents (63.8% girls) aged 13–20 years (M 16.54, SD 0.88) with BMIs ranging from 12.63 to 24.89 (M 22.57, SD 1.98) were included in the analyses.

### Procedure

The study was conducted in 16 public middle and high schools in Central and Eastern Poland. For all participants T2 data were collected at 11 months after T1. All potential respondents lived with their parents (98.9%) or other legal guardians (1.1%) at T1 and T2. Participants and parents of individuals younger than 18 years provided informed consent before the study. Adolescents were informed about the objectives, the procedure and the possibility of refusing to participate in the study (in this case access to school’s library or dayroom was provided). Personal codes were assigned to secure anonymity and identification across the measurement points. Participants were asked to provide their data referring to their nutrition behaviors and body satisfaction. At T1, participants completed a questionnaire measuring their body satisfaction, and at T2 participants (70.2% of those who completed T1) filled in a questionnaire regarding their eating behaviors. At both T1 and T2, participants have their height and weight measured. Researchers were available for consultations after the study completion and made multiple efforts to reduce attrition. The study was approved by the Institutional Review Board and Ethics Committee at University of Social Sciences and Humanities, Poland. The procedures of the study were described in more detail in a paper by Zarychta et al. [10].

### Materials

The hypotheses in the present study are tested using two measurement points to establish temporal precedence [29]. Thus, the independent variable was measured at T1, and the mediators and dependent variable at T2.

#### Body satisfaction (T1)

Body satisfaction variable consisted of seven items, based on The Multidimensional Body-Self Relations Questionnaire’s Appearance Evaluation Subscale (MBSRQ; Cash [38]). The measure assesses feelings of physical attractiveness or unattractiveness with high scores indicating higher satisfaction with one’s appearance and low scores indicating general unhappiness with one’s appearance. In order to assess it, the respondents were asked to read seven statements (e.g., “I like my looks just the way they are”, “Most people would consider me good-looking” and “I like the way I look without my clothes on”) and decide how much each statement pertains to them. The responses ranged from 1 (*definitely disagree*) to 5 (*definitely agree*).

#### Healthy eating (T2)

In order to evaluate healthy eating, two questions adopted from Lally, Bartle and Wardle [39] were used: “How often did you eat a portion of fresh fruit in the last two weeks?” and “How often did you eat a portion of vegetables in the last two weeks (fresh, boiled or fried without fat)?”. The portion was defined as the amount fitting into a cupped hand. The responses were given on a 6-point scale, ranging from 1 (*once a week or less*) to 6 (*four or more times a day*).

#### Unhealthy eating (T2)

In order to evaluate unhealthy eating, adolescents answered two questions, adopted from Lally, Bartle and Wardle [39]: “How often did you eat fatty foods (e.g., pizza, chips, foods with dressings) in the last two weeks?” and “How often did you eat sweets (e.g., chocolate bars or wafers, cakes) in the last two weeks?”. The responses were given on a 6-point scale, ranging from 1 (*once a week or less*) to 6 (*four or more times a day*).

#### Restrictive dieting (T2)

In line with research conducted so far on overall diet quality (cf. Loftfield et al. [40]), the measurement of dietary restrictions was based on a single item adopted from MBSRQ [38]: “I am on a restrictive weight-loss diet”. The responses ranged from 1 (*definitely disagree*) to 5 (*definitely agree*).

#### Body weight and height (T1 and T2)

Biometric measures were assessed with standard medically approved telescopic height measuring rods and floor scales (scale type: BF-100 or BF-25). Age- and sex-specific BMI percentiles were calculated with WHO AnthroPlus macro [41], based on the WHO growth reference [37] for children and adolescents. BMI *z*-scores were calculated and used as independent variables in both analyses. Also, two weight status categories were created based on BMIs’ SD cut-offs (− 1—underweight [less than or equal to 2 SD], + 1—normal weight) [37].

### Data analysis

**Fig. 1:**
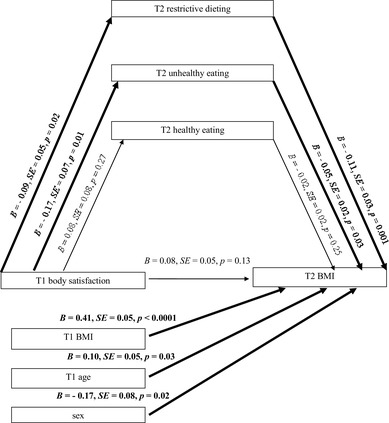
The mediating effects of restrictive dieting, unhealthy eating, and healthy eating. *T1* Time 1, baseline; *T2* Time 2, 11-month follow-up, *BMI* body mass index *z*-scores. Paths marked in bold represent significant associations

Data were analyzed using SPSS version 24. Multiple mediation analysis was performed to test the relation between body satisfaction, healthy eating, unhealthy eating or restrictive dieting, and adolescents’ BMIs with the use of PROCESS macro (Model 4) with 10,000 bootstraps [42]. Analyses were conducted accounting for the covariates (T1 BMI, age and sex [coded as 1 for males and 2 for females]). Results are presented using two types of coefficients: (1) the regression coefficient for each parameter (see Fig. 1), and (2) the indirect effect coefficient (*B*) for each indirect pathway between the independent variable (T1 body satisfaction) and the dependent variable (T2 BMI), accounting for respective mediators (see Fig. 1; Table 2). Furthermore, sex, age, and BMI (T1) were included into each regression model as predictors of the dependent variable, BMI at T2. This way potential confounders and BMI at T1 were controlled for.

Additionally, moderated multiple mediation analysis was conducted using PROCESS macro (Model 58). In particular, these analyses tested if the associations between (1) the independent variable and the mediator, and (2) the mediator and the dependent variable are moderated by participants’ weight status.

In the current study the independent variable (IV) was body satisfaction (T1); the dependent variable (DV) was BMI *z*-score measured at T2; the mediators were healthy eating (T2), unhealthy eating (T2) and restrictive dieting (T2); moderator was weight status (T1) coded as − 1 for underweight and + 1 for normal body weight.

In order to establish temporal precedence [29], the variables were measured at different time points (T1 and T2). Missing data were imputed with multiple imputation method [43]. A total of 5.9% of the completers’ data were missing. The attrition analysis is presented below.

## Results

### Preliminary analyses

#### Descriptive findings and attrition analysis

Means, standard deviations, results of variance analyses and reliability coefficients are presented in Table 1.

**Table 1:** Descriptive statistics, reliability, and correlations between the study variables at T1 and T2 (*N* = 1042)

		Total sample: M (SD)	*α*	2	3	4	5	6	7	8	Differences between underweight and normal weight group: *F*	M (SD) for underweight/M (SD) for normal weight group	Differences between males and females: *F*	M (SD) for males/M (SD) for females
1	T1 Body satisfaction	2.54 (0.72)	0.77	0.04	0.08*	− 0.07*	0.06*	0.08**	− 0.01	0.11***	1.31	2.33 (0.55)/ 2.53 (0.73)	2.25	2.41 (0.65)/ 2.59 (0.77)
2	T2 Healthy eating	5.21 (1.68)	0.71		0.06^†^	− 0.12***	0.02	− 0.01	0.01	0.01	0.87	5.00 (1.37)/ 5.20 (1.69)	0.89	5.14 (1.74)/ 5.22 (1.65)
3	T2 Unhealthy eating	4.49 (1.80)	0.58			− 0.02	− 0.09*	− 0.09*	− 0.02	− 0.07**	2.12	3.95 (1.18)/ 4.32 (1.54)	0.84	4.44 (1.55)/ 4.23 (1.52)
4	T2 Restrictive dieting	4.00 (1.13)					− 0.04*	− 0.14***	− 0.04	− 0.01*	0.56	4.00 (1.25)/ 4.00 (1.13)	0.44	4.00 (1.13)/ 4.00 (1.14)
5	T1 BMI	0.04 (0.94)						0.23***	0.04	− 0.15***	8.47***	− 0.44 (0.54)/ − 0.12 (0.69)	4.92***	− 0.05 (0.67)/ − 0.24 (0.779)
6	T2 BMI	0.14 (1.15)							0.06*	− 0.11**	6.83***	− 0.37 (1.25)/ − 0.12 (1.15)	2.38***	0.03 (1.09)/ 0.02 (1.17)
7	T1 Age	16.41 (0.77)								− 0.02	0.34	16.74 (0.65)/ 16.53 (0.87)	0.58	16.41 (0.94)/ 16.38 (0.66)

*T1* Time 1, baseline, *T2* Time 2, 11-month follow-up, *BMI* body mass index *z*-scores

***p < 0.001; **p < 0.01; *p < 0.05; ^†^*p* < 0.1

Completers did not differ from those who dropped out at T2 in terms of the body satisfaction, healthy eating, unhealthy eating, restrictive dieting and BMI, all *F*s < 3.21, *p*s > 0.15, or sex, χ2 (1) = 2.70, *p* = 0.14. Dropouts and completers differed in terms of age, *F* (1, 1296) = 62.17, *p* < 0.0001 with dropouts being slightly older (M 16.82, SD 0.64) than completers (M 16.40, SD 0.98, Cohen’s *d* = 0.50 [95% CI 0.46–0.55]).

#### Correlation analyses and comparisons between adolescents with underweight and normal body weight

Correlations between study variables for the study sample (*N* = 1042) are presented in Table 1. There were weak negative correlations between body satisfaction (T1) with restrictive dieting (T1 and T2), adolescents’ BMI (T1 and T2), their age and sex. Healthy eating (T1 and T2) was weakly and negatively associated with restrictive dieting (T1 and T2). On the other hand, unhealthy eating (T1 and T2) was weakly and positively associated with restrictive dieting (T1 and T2). Moreover, restrictive dieting (T1 and T2) was weakly and negatively correlated with adolescents’ BMI (T1 and T2), and their sex (see Table 1).

No significant differences were found between adolescents with underweight and normal body weight in terms of body satisfaction (T1), healthy eating (T2), unhealthy eating (T2) or restrictive dieting (T2), all *F*s < 2.12, *p*s > 0.15 (see Table 1).

### Indirect effect of body satisfaction mediated by healthy eating

For the first three hypotheses one multiple mediation model (Model 4, which allows up to 10 mediators operating in parallel) was conducted.

H1 tested the indirect effect of *body satisfaction* (T1) (IV) on adolescents’ BMI (T2) (DV) mediated by *healthy eating* (T2) (see Fig. 1). It was measured controlling for BMI (T1), adolescents’ sex and age.

The results of multiple mediation analysis for H1 (see Table 2) indicated no significant indirect effect of *body satisfaction* (T1) on adolescents’ BMI (T2). Also, we did not find any direct associations between these three variables. Additional moderation analysis, assuming that the associations between body satisfaction, healthy eating, and adolescents’ BMI were moderated by sex, indicated that these associations were the same for girls and boys.

**Table 2:** Indirect effects of body satisfaction on BMI

Indirect effects pathways			*B*	SE	BC 95% CI	
					Lower	Higher
*Testing the indirect effect of body satisfaction*						
H1	Body satisfaction T1 → healthy eating T2 → BMI T2		− 0.002	0.003	− 0.012	0.001
**H2**	**Body satisfaction T1** → **unhealthy eating T2** → **BMI T2**		**0.009**	**0.005**	**0.001**	**0.023**
**H3**	**Body satisfaction T1** → **restrictive dieting T2** → **BMI T2**		**0.010**	**0.007**	**0.001**	**0.026**

Indirect effects pathways	Moderated mediation index	SE	BC 95% CI	
			Lower	Higher
*Testing the moderated role of body weight status (H4)*				
Body weight status moderating body satisfaction T1 → healthy eating T2	− 1.30	0.72	− 2.72	0.12
Body weight status moderating body satisfaction T1 → unhealthy eating T2	0.13	0.66	− 1.16	1.43
Body weight status moderating body satisfaction T1 → restrictive dieting T2	0.01	0.49	− 0.94	0.97
Body weight status moderating healthy eating T2 → BMI T2	− 0.22	0.23	− 0.67	0.23
Body weight status moderating unhealthy eating T2 → BMI T2	0.10	0.23	− 0.34	0.54
Body weight status moderating restrictive dieting T2 → BMI T2	0.44	0.21	− 0.03	0.85

Values of indirect effect coefficient (*B*) presented in bold are significant. Each bootstrap was based on 10,000 repetitions. Bias corrected (BC) confidence intervals (CI) that do not include zero indicate a significant indirect effect

Significant coefficients are marked in bold

*T1* Time 1, baseline, *T2* Time 2, 11-month follow-up, *H* Hypothesis, *BMI* body mass index *z*-scores

### Indirect effect of body satisfaction mediated by restrictive dieting

H2 tested the indirect effect of *body satisfaction* (T1) (IV) on adolescents’ BMI (T2) (DV) mediated by *unhealthy eating* (T2) (see Fig. 1). Analysis was conducted controlling for BMI (T1), adolescents’ sex and age.

The results of multiple mediation analysis for H2 (Table 2) showed that the association between *body satisfaction* (T1) and adolescents’ BMI (T2) was mediated by *unhealthy eating* (T2) as indicated by the significant indirect effect. Adolescents who were unhappy with their appearance (T1) eat unhealthy food more often (T2) and predicted lower BMI at T2. Direct association between IV (T1), mediator (T2), and DV (T2) is presented in the Fig. 1.

### Indirect effect of body satisfaction mediated by restrictive dieting

H3 tested the indirect effect of *body satisfaction* (T1) (IV) on adolescents’ BMI (T2) (DV) mediated by *restrictive dieting* (T2) (see Fig. 1). Analysis was conducted controlling for BMI (T1), adolescents’ sex and age.

The results of multiple mediation analysis for H3 (Table 2) showed that the association between *body satisfaction* (T1) and adolescents’ BMI (T2) was mediated by *restrictive dieting* (T2) as indicated by the significant indirect effect. Adolescents who were unhappy with their appearance (T1) were also dieting restrictively more often (T2) and thus had lower BMI at T2. Direct association between IV (T1), mediator (T2), and DV (T2) is presented in the Fig. 1. The analyses assumed that the associations between body satisfaction, restrictive dieting, and adolescents’ BMI were moderated by sex, indicated that these associations were the same for girls and boys.

### Testing the moderating role of body weight status

To test whether *body weight status* act as a moderator of the associations between (1) *body satisfaction* (T1) (IV) and *healthy eating* (T2), *unhealthy eating* (T2) and *restrictive dieting* (T2) (mediators), and (2) between mediators (T2) and BMI (T2) (DV), moderated multiple mediation analysis (Model 58, which allows up to 10 mediators operating in parallel) was conducted controlling for BMI (T1), adolescents’ sex and age.

The results of moderated multiple mediation analysis (Table 2) showed no significant effects of *body weight status* on any of above-mentioned associations. Additional multiple moderation analysis, assuming that the associations between body satisfaction, unhealthy eating, and adolescents’ BMI were moderated by sex, indicated that these associations were the same for girls and boys.

## Discussion

This study provides novel evidence for the long-term indirect effects of body satisfaction on adolescents’ BMI through restrictive dieting, healthy and unhealthy eating in the non-clinical group of under- and healthy-weight adolescents. The results show that body satisfaction is indirectly linked to adolescents’ BMIs through restrictive dieting but also through unhealthy eating. However, the mediating effects were not found for healthy eating. In particular, lower body satisfaction at T1 predicted more unhealthy eating and restrictive dieting at T2 which in turn predicted lower BMI at T2.

In contrast to previous cross-sectional research (e.g., Tylka [13]) or research which explored direct associations between variables presented in our study (e.g., Mendonça et al. [23]), this research permitted a more thorough testing of associations between body satisfaction, healthy and unhealthy eating, restrictive dieting and BMI in the non-clinical group of under- and healthy-weight adolescents. Thus, our research expands on previous research conducted in general population of adolescents [26] or only in groups of overweight and obese adolescents [27, 28], and shows that these associations are true also across under- and healthy-weight adolescents and in a large sample of both male and female participants. Moreover, we found no significant moderating effect of sex, which means that associations between study variables were the same for both sexes. This is congruent with previous studies (e.g., Field et al. [22]) that indicated that both females and males might behave similarly in terms of eating, but the purposes of such behaviors might be different. For girls it is having a lean body, and for boys it is having lean and muscular body.

Previous research [18, 44] confirmed that body satisfaction mattered, being a predictor of dieting and other types of weight control behaviors or binge eating. However, no studies tested if body satisfaction predicts healthy or unhealthy eating behaviors, which in turn predicts unfavorable changes in BMI. The present study filled that gap indicating that body satisfaction is not only a predictor of restrictive dieting, but also of unhealthy eating, and both these associations mediate the relationship between body satisfaction and adolescents’ BMIs. Thus, theoretical models analyzing body satisfaction role (e.g., Fairburn [6]) should account not only for dietary restrictions but also for energy-dense eating.

The main limitation of the study refers to an exclusion of affective and other cognitive processes which explain ED symptoms and body weight according to cognitive-behavioral models. Assuming small effects of cognitions and behaviors on changes in body mass (T1–T2), an addition of further emotional, cognitive and behavioral processes would require a larger sample to secure good power of analyses. Moreover, it has to be taken into account that the outcomes may be affected by social desirability bias and the composition of the study sample, i.e., a non-clinical sample of under- and healthy-weight adolescents. This study aimed at indicating potential mechanisms of developing ED (and lower body weight as one of its main symptoms), though. Therefore, a clinical sample of patients with ED has been found to be inappropriate to achieve this aim. Yet, there was no screening for ED symptoms performed in this study. Thus, the results may refer to a general population including both adolescents with and without ED symptoms. Another limitation are low beta coefficients obtained in this study indicating weak associations between the variables and in consequence the results should be interpreted cautiously. Reliability of the single-item measure of dieting may be limited, but its usage has been justified in previous research [e.g., 40]. Alternatives may be considered in the future, though. Moreover, body weight depends on eating behaviors but also on energy expenditure levels, that is physical activity. Thus, to fully understand the links between body satisfaction and body weight, both eating behaviors and physical activity should be accounted for in future research. Finally, we did not account for other factors previously found to be significant for the association between body satisfaction and ED symptoms, such as negative affect [45], depressed mood [46], emotion regulation [47], gender differences and personality traits [48], maladaptive perfectionism [49], self-esteem [50] or socio-economic status [51].

### Conclusions

In conclusion, the results of this study confirmed associations between body satisfaction, unhealthy eating and restrictive dieting and adolescents’ BMIs, specifically in the group of under- and healthy-weight adolescents. These results indicate that the inclusion of cognitive factors such as body satisfaction, and behavioral symptoms of ED such as unhealthy eating and restrictive dieting in most treatment and prevention programs of ED is justified and should continue. Our findings, however, suggest that ED prevention or treatment programs might target not only adolescents from clinical samples, but also under- and healthy-weight adolescents who are at risk of unhealthy eating and at the same time dieting restrictively, and who are highly dissatisfied with their bodies, especially.
